# Therapeutics

**Published:** 1880-11

**Authors:** 


### THERAPEUTICS.

THERAPEUTIC USE OF IODOFORM.—(*Jour. de Méd.* p. 371, Aug., 1880.)

According to Dr. Lindemann, the balsam of Peru completely masks the odor of iodoform; two parts of the balsam completely neutralize one part of iodoform. The best vehicles are lard, glycerine, or better still, vaseline. Here is a formula which the author recommends:

Iodoform........................................1 part.
Balsam of Peru.................................3 parts.
Vaseline ........................................8 parts.

He also often prescribes the following:

Iodoform........................................1 part.
Balsam of Peru.................................3 parts.
Alcohol, glycerine or collodium............12 parts.

First mix the iodoform and balsam thoroughly, then add the other ingredients.

---

THE USE OF PURE WATER IN ERECTING BUILDINGS. Dr. Walter Fergus writes, in the *Lancet* of October 9, and certainly his suggestion is well worthy of consideration: In reading your remarks upon the prosecution of the builders who used earth in place of mortar at Edmonton, it occurred to me to mention how important it is to use pure water in mixing mortar, whether for building or for plastering dwelling-houses. Newly-built houses are notoriously apt to prove anything but salubrious to the first inmates. Much of this unhealthiness of new houses may be owing to the use of foul and polluted water for making the mortar.

A NEW and apparently a most valuable method of preserving raw meat, discovered by Prof. Artimini, of Florence, and patented in this country, bids fair to supply a long-felt want and to have an appreciable effect on our markets. According to a report by Profs. Barff and Mills, of Glasgow, and Dr. Stevenson, of Guy's Hospital, meat six months old was found to be perfectly sound and good, the muscular fiber unchanged, and the nutritive properties unimpaired (*Med. Times and Gazette*). The material employed is stated to be less expensive than salt, and not only wholesome, but pleasant to the taste.

